# Simultaneous Bilateral Neck of Femur Fracture in a Young Adult with Underlying Metabolic Disturbances

**DOI:** 10.1155/2020/8972542

**Published:** 2020-01-29

**Authors:** Eslam Alkaramani, Motasem Salameh, Mohammed Adam, Bivin George, Yaser Alser, Ghalib Ahmed

**Affiliations:** Orthopedic Surgery Department, Hamad General Hospital, Doha, Qatar

## Abstract

*Introduction*. Simultaneous bilateral fractures of the femoral neck are considered very rare injuries. Few cases were reported in the literature. Most cases were reported in elderly patients with underlying bone pathology. *Case Report*. We report a case of a 31-year-old male patient who presented to the emergency department with bilateral hip pain and inability to bear weight after a sudden loss of consciousness and fall while running on a treadmill. The patient had a recent history of anabolic steroids, growth hormone, and other supplements used for bodybuilding. Radiological studies confirmed bilateral neck of femur fracture. Laboratory investigations revealed pan-pituitary axis insufficiency and mild vitamin D deficiency, and his EEG suggested a seizure attack. The patient was treated with three 6.5 mm cannulated cancellous screws on one side and a sliding hip screw on the other side and was followed with strict physical therapy and rehabilitation plan. 6 months from the injury, a radiographic bilateral union achieved with the patient back to his normal daily activity and noncontact sports.

**Conclusion:**

We report this rare case of bilateral neck of femur fracture in a young adult after a generalized seizure attack with underlying metabolic disturbances. Ruling out other biological underlying etiologies, early diagnosis and early fracture anatomic reduction and fixation are crucial to decrease potential complications such as avascular necrosis and fracture nonunion.

## 1. Introduction

Femoral neck fractures in adults younger than 50 represent 2-3% of all neck of femur fractures [1]. Most of these fractures in this age group are caused by high-energy trauma [1, 2]. Simultaneous atraumatic bilateral femoral neck fractures in young adults are considered very rare injuries. Few cases were reported in literature in this age group [3–9]. These fractures are associated with complications such as avascular necrosis and nonunion [10, 11]. Anatomical reduction and early fixation and rehabilitation are crucial to avoid such devastating complications. We report this rare case of bilateral neck of femur fracture in a young adult after a grand mal seizure combined with metabolic disturbances.

## 2. Case Report

A 31-year-old male—previously healthy—was brought to the emergency department by ambulance after he lost his consciousness and fell down during running on a treadmill with no witnesses to the event. He was complaining of bilateral hip pain and inability to bear weight. The patient denied any incontinence but reported tongue biting. The patient had a history of drug abuse for body building purposes for the past 3 years. He reported taking anabolic steroids, growth hormone, thyroxine, and creatinine with no professional supervision and no compliance for dose limits.

**Figure 1:**
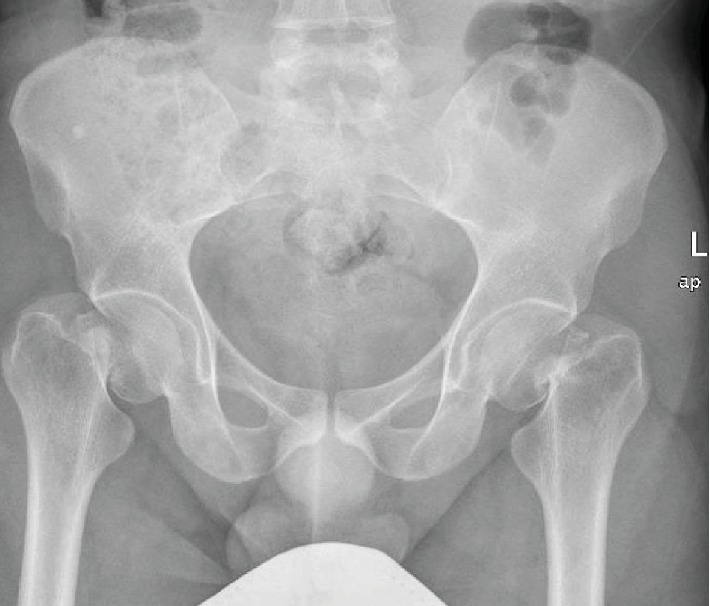
Anteroposterior pelvis radiograph showing the bilateral Garden 4 neck of femur fractures.

**Figure 2:**
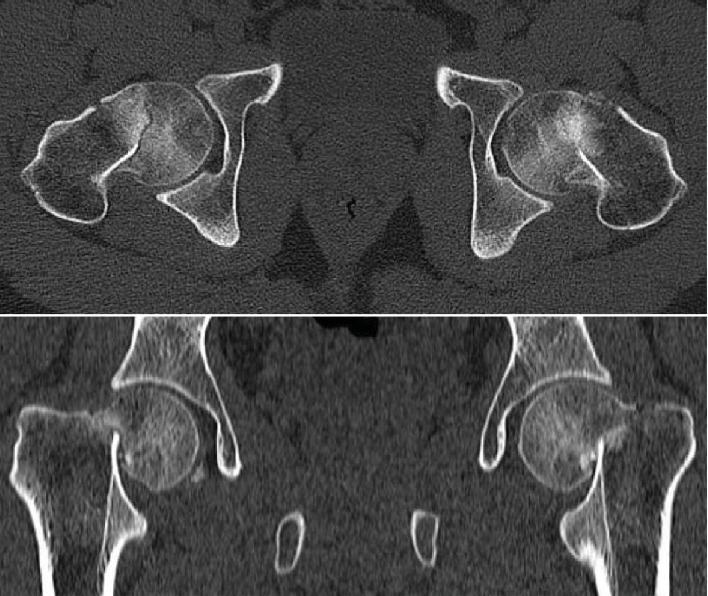
Axial and coronal pelvis CT scan cuts showing bilateral neck of femur fracture.

Upon physical examination, the patient was confused, with tender bilateral hips and externally rotated lower limbs, with no neurovascular compromise. Laboratory investigations revealed pan-pituitary axis insufficiency and mild vitamin D deficiency (Table 1). Radiological investigations showed bilateral neck of femur fractures. Both of which were graded as type IV according to Garden's classification (Figure 1). A computed tomography (CT) scan of the pelvis confirmed the diagnosis with more comminution seen in the left side (Figure 2). A CT scan of the head was done and was unremarkable. Endocrinologists were consulted, and the advice was to keep the patient on corticosteroids and wean him off after the surgical intervention.

**Figure 3:**
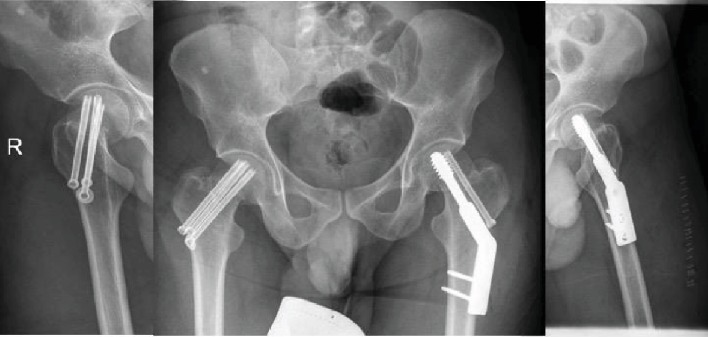
Immediate postoperative pelvis and hip radiographs.

The patient was stabilized and cleared for surgical intervention. He was operated on the same day of admission. A fracture table was used to facilitate closed reduction. After sound reduction fixation was achieved by 6.5 mm cannulated cancellous screws on one side and a sliding hip screw with an antirotation screw on the other side. The senior author's decision to fix the left side with a sliding hip screw was explained by more comminution and higher risk of construct failure compared to the right side. Immediate postoperative images showed acceptable reduction and fixation (Figure 3). Later during the admission, the neurology team was consulted and an Electroencephalogram (EEG) showed Frontal Intermittent Rhythmic Delta Activity (FIRDA), and Magnetic Resonance Image (MRI) of the brain was unremarkable. The patient was diagnosed as a case of Generalized Tonic Clonic Seizure (GTCS) and was started on levetiracetam.

**Figure 4:**
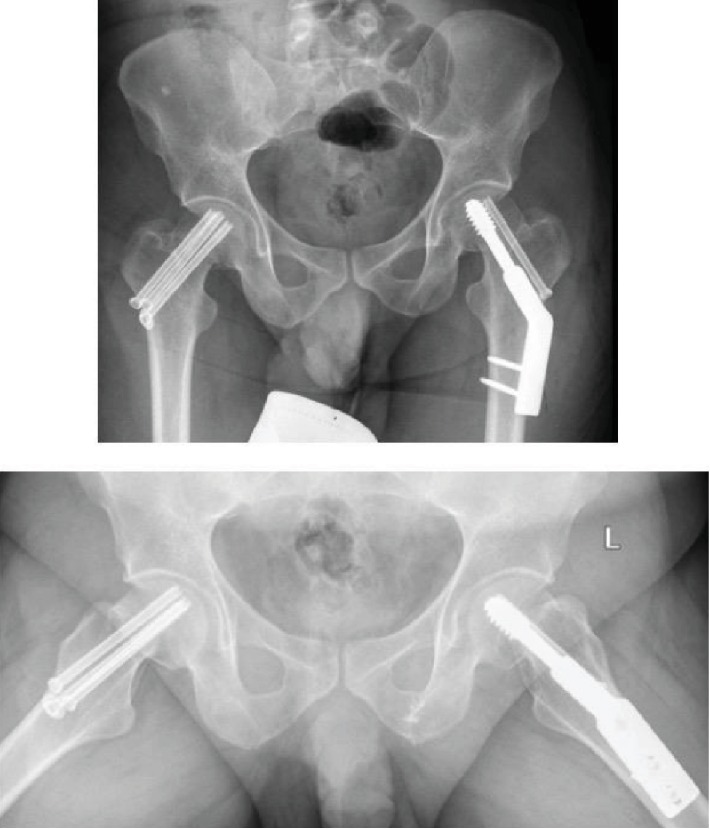
18-month follow-up images showing radiographic union of both sides with no signs of avascular necrosis.

The patient was discharged on a wheel chair at the beginning and gradually converted to partial- and then full-weight bearing within four months. He had a total of six months of regular follow-up postoperatively with strict physical therapy and rehabilitation plan. In the last follow-up 18 months postinjury, a plain radiograph showed complete fracture union on both sides with no signs of avascular necrosis (Figure 4). His gait was normal, and he could return back to his normal daily activity and noncontact sports.

A follow-up EEG and video monitoring after stopping the hormones and supplements for 6 months were unremarkable, the final diagnosis was a single episode of GCTS due to an overdose of anabolic hormones, and the epilepsy medication was stopped by the neurologist.

## 3. Discussion

We presented this rare case of a 31-year-old healthy male patient with simultaneous atraumatic bilateral neck of femur fractures, and this type of injury in this age group was reported in the literature in few case reports and was the result of high-energy trauma, seizure activity [3, 4, 7], electrical shock [9], or altered bone metabolism [5, 6, 8].

Our case was diagnosed with GTCS with abnormal EEG with no detectable brain lesions on imaging studies, and FIRDA was linked with metabolic disturbances [12, 13] in neurophysiological studies. This would explain this abnormal activity in our patient's EEG who had pan-hypopituitarism due to hormonal abuse for his weight loss and bodybuilding regimen.

The strong muscle contractions during seizure attacks can cause fractures and dislocations with a rate of 1% [14]. Bilateral neck of femur fractures compromised 6% of the fractures that occur after generalized seizures [15]. Nevertheless, sustaining bilateral neck of femur fracture after a single convulsion should raise the suspicion of underlying bone disease. Our case had a mild deficiency in vitamin D which is endemic in our region, and the other significant finding was the inhibition of his hypothalamic-pituitary access. We believe that the patient's vitamin D deficiency and the concurrent use of steroids altered his bone metabolism and rendered his bone weaker.

Cagirmaz et al. [7] reported a similar case of a 24-year-old male that was treated with bilateral closed reduction and percutaneous screw fixation; postoperatively, the patient was diagnosed with osteopenia with −1.9 T score in the lumbar spine. Shah et al. [4] reported a simultaneous bilateral neck of femur fracture after a hypoglycemic seizure attack in a 30-year-old male, with both sides fixed closed with percutaneous screws.

In conclusion, we present this case of a young adult with atraumatic bilateral neck of femur fracture after a tonic clonic seizure with underlying metabolic bone disease. Ruling out underlying biological etiology, early diagnosis and early fracture anatomic reduction and fixation are crucial in the management of bilateral neck of femur fractures.

## Figures and Tables

**Table 1:** Patient's lab results on the day of admission.

Lab	Result	Normal hospital range
Vitamin D	19 ng/ml	10–30 ng/ml—mild-to-moderate deficiency
PTH—plasma	149 pg/ml	15–65 pg/ml
Calcium corrected	2.05 mmol/l	2.10–2.55 mmol/l
ACTH	<2 pg/ml	5–60 pg/ml
Cortisol	<22 nmol/l	138–580 nmol/l
SHBG	9.0 nmol/l	10.0–55.0 nmol/l
FSH	0.20 IU/ml	1.00–19.00 IU/ml
LH	<0.5 IU/l	1.0–9.0 IU/l
Testosterone	1.06 nmol/l	10.40–35.00 nmol/l
TSH	0.35 mIU/l	0.45–4.50 mIU/l
FT3	2.32 pmol/l	2.89–4.88 pmol/l
FT4	9.7 pmol/l	9.0–20.0 pmol/l

PTH = parathyroid hormone; ACTH = adrenocorticotropic hormone; SHBG = sex hormone-binding globulin; FSH = follicular-stimulating hormone; LH = luteinizing hormone; TSH = thyroid-stimulating hormone; FT3 = free triiodothyronine; FT4 = free thyroxine.
